# Daikenchuto accelerates the recovery from prolonged postoperative ileus after open abdominal surgery: a subgroup analysis of three randomized controlled trials

**DOI:** 10.1007/s00595-019-01787-9

**Published:** 2019-02-25

**Authors:** Toru Kono, Mitsuo Shimada, Masaaki Nishi, Yuji Morine, Kozo Yoshikawa, Hidetoshi Katsuno, Koutarou Maeda, Keisuke Koeda, Satoshi Morita, Masahiko Watanabe, Mitsuo Kusano, Junichi Sakamoto, Shigetoyo Saji, Hiroki Sokuoka, Yasuto Sato, Yoshihiko Maehara, Takashi Kanematsu, Masaki Kitajima

**Affiliations:** 1 Advanced Surgery Center, Sapporo Higashi Tokushukai Hospital, 3-1, N 33, E 14, Higashi-ku, Sapporo, Hokkaido 065-0033 Japan; 2 Department of Digestive Surgery and Transplantation, Institute of Biomedical Sciences, Tokushima University Graduate School of Medicine, Tokushima, Japan; 3 Department of Surgery, School of Medicine, Fujita Health University, Toyoake, Japan; 4 International Medical Center, Fujita Health University Hospital, Toyoake, Japan; 5 Department of Medical Safety Science, School of Medicine, Iwate Medical University, Morioka, Japan; 6 Department of Biomedical Statistics and Bioinformatics, Kyoto University Graduate School of Medicine, Kyoto, Japan; 7 Department of Surgery, Kitasato University School of Medicine, Sagamihara, Japan; 8 Department of Physical Medicine and Rehabilitation, Ohta General Hospital Foundation Ohta Atami Hospital, Koriyama, Japan; 9 Tokai Central Hospital, Kakamigahara, Japan; 10 Public Interest Incorporated Foundation, Japanese Foundation for Multidisciplinary Treatment of Cancer, Koto-ku, Japan; 11 Department of Public Health, Tokyo Women’s Medical University, Shinjuku-ku, Japan; 12 Nagasaki City Hospital Organization, Nagasaki, Japan; 13 International University of Health and Welfare, Minato-ku, Japan

**Keywords:** Kampo, Body mass index, Postoperative ileus

## Abstract

**Purpose:**

Prolonged postoperative ileus (POI) is a common complication after open abdominal surgery (OAS). Daikenchuto (DKT), a traditional Japanese medicine that peripherally stimulates the neurogenic pathway, is used to treat prolonged POI in Japan. To analyze whether DKT accelerates the recovery from prolonged POI after OAS, we conducted a secondary analysis of three multicenter randomized controlled trials (RCTs).

**Methods:**

A secondary analysis of the three RCTs supported by the Japanese Foundation for Multidisciplinary Treatment of Cancer (project numbers 39-0902, 40-1001, 42-1002) assessing the effect of DKT on prolonged POI in patients who had undergone OAS for colon, liver, or gastric cancer was performed. The subgroup included 410 patients with no bowel movement (BM) before the first diet, a DKT group (*n* = 214), and a placebo group (*n* = 196). Patients received either 5 g DKT or a placebo orally, three times a day. The primary endpoint was defined as the time from the end of surgery to the first bowel movement (FBM). A sensitivity analysis was also performed on the age, body mass index and dosage as subgroup analyses.

**Results:**

The primary endpoint was significantly accelerated in the DKT group compared with the placebo group (*p* = 0.004; hazard ratio 1.337). The median time to the FBM was 113.8 h in the placebo group and 99.1 h in the DKT treatment group.

**Conclusions:**

The subgroup analysis showed that DKT significantly accelerated the recovery from prolonged POI following OAS.

**Trial registration number:**

UMIN000026292.

## Introduction

Postoperative ileus (POI), which is a routine and unavoidable consequence of major open abdominal surgery (OAS), is characterized by transient impairment of bowel motility [1–3]. Patients suffering from POI in the small and large intestines commonly recover 24–48 and 48–72 h after surgery, respectively [3, 4]. Therefore, uncomplicated “normal” POI that resolves spontaneously within 2–3 days after surgery is considered to be a physiological event [3, 4]. By contrast, prolonged POI, which continues postoperatively beyond day 4, is termed “pathologic POI” [1, 5]. Several mechanisms have been proposed to explain the pathogenesis of prolonged POI, especially that after OAS, including a disturbance of the neurogenic pathway [5, 6]. Although several advances have been made in medical therapy to help reduce the incidence and severity of POI [5, 6], the number of people suffering with this condition nonetheless remains high.

Daikenchuto (DKT), which is a mixture of extract powders from dried Japanese pepper, processed ginger and ginseng radix, is a scientifically validated and frequently prescribed traditional Japanese Kampo medicine [7, 8]. Kampo medicines meet the strict specifications for coverage under the Japanese National Health Insurance plan, which is comparable to Western counterparts in terms of ensuring the quality and therapeutic efficacy. Furthermore, DKT, which is known to stimulate the enteric and sensory neural pathways in the intestinal wall, has been approved as an investigational drug by the US Food and Drug Administration. DKT is primarily used for the treatment of POI [7–9]. To gather clinical evidence, three randomized controlled trials (RCTs) supported by the Japanese Foundation for Multidisciplinary Treatment of Cancer (JFMC; project numbers 39-0902, 40-1001, 42-1002) were conducted to determine whether or not DKT ameliorated POI [10–12]. The results of one of the RCTs indicated a significant decrease in time until the first passage of stool [11]. The remaining two RCTs showed a slight tendency toward amelioration of POI [10, 12].

Although there is a lack of consensus regarding the “normal” interval for distinguishing pathologic POI from physiologic POI, a proposal and validation demonstrated the best endpoint to define pathologic POI to be the combination of the passage of stool and tolerance of solid food [1, 13].

Defecation [passing a bowel movement (BM)] is the ultimate indicator of the intestinal motility. More than half of patients were reported to spontaneously recover from POI within three to four days (physiologic POI) after major laparotomy surgery, as determined by the resumption of BM [1, 13]. These patients had already recovered from physiologic POI, and since the incidence of pathologic POI is low, it was believed that recurrent POI would be unlikely to occur again. We therefore hypothesize that the effect of DKT on “pathologic POI” might differ after excluding such low-risk patients.

In the present study, we investigated the effect of DKT on POI in patients with no BM before the first diet after OAS. Three RCTs were conducted without DKT pharmacokinetic data. A recent study showed that the concentrations of active DKT ingredients that enter the plasma may be lower in older patients than in younger ones and in those with a high body mass index (BMI) than in those with a low BMI [14]. The practical dosage of DKT is also a direct factor affecting the concentrations of active DKT ingredients. Thus, subpopulations extracted by several conditions based on the values of these variables were used. Additional subpopulations used in the sensitivity analyses included patients with no remarkable medical history, coexisting diseases, or surgery-related complications.

## Methods

### Ethics

This study was conducted in accordance with the tenets of the Declaration of Helsinki and was approved by the internal review boards of all participating institutions. This pooled analysis study was approved by the institutional review board of the JFMC.

### Clinical trials and subgroup assignment

The three clinical trials (JFMC project numbers 39-0902, 40-1001, and 42-1002) were performed to evaluate the clinical benefits of DKT in POI after major OAS for cancer of the colon, liver, or stomach. These studies were planned by the study group “DKT Forum” consisting of surgeons in the different fields from 33 academic medical centers (22% of all academic medical centers in Japan), and the protocols were standardized as much as possible to enable pooled/integrated post-analyses.

A total of 862 enrolled patients were randomized to receive either 5 g DKT or placebo orally, three times a day, for a maximum of 12 days. The patients were observed for up to 14 days after surgery. Of these 862 patients, 122 were excluded for the following reasons [JFMC39 (colon); 50 excluded] 32 patients were considered ineligible for the study (8 had stoma; 7 had stage IV cancer; 5 had a history of laparotomy; 4 had complications, including ileus or hepatic disease; 3 were not diagnosed with colon cancer; 1 underwent laparoscopic surgery; 1 declined to give informed consent; 1 had double cancer; and 2 were dismissed for other reasons), and 18 patients were not treated (7 could not be treated because of their circumstances, 5 suffered complications, 2 were intolerant to drug administration, 2 refused drug administration, 1 declined treatment after initiation of the study, and 1 could not receive treatment because of his conditions); [JFMC40 (liver); 22 excluded] 15 patients were considered ineligible for the study (4 in the DKT group and 11 in the placebo group; specifically, 7 did not receive surgery, 2 were unresectable cases, and 6 received chemotherapy within 6 weeks), and 7 in the DKT group were untreated (2 withdrew their consent, 1 rejected the treatment, 2 were non-cancer patients, and 2 had their date of operation changed); [JFMC42 (stomach); 50 excluded] 38 patients were considered ineligible for the study (19 in the DKT group and 19 in the placebo group; specifically, 22 did not undergo open total gastrectomy, 1 did not have surgery, 3 had a history of laparotomy, 7 had another intestinal resection at the time of gastrectomy, 1 received chemotherapy in the preceding 4 weeks, and 4 withdrew their consent), and 12 were not treated (1 each declined treatment, had anastomotic leakage, showed difficulty with oral intake, and had a liver functional disorder; 7 were not treated by the doctor’s recommendation, and 1 started DKT at postoperative day 3).

Among all patients enrolled and randomized in the 3 RCTs, 740 patients who were eligible were ultimately included in the efficacy analysis.

### Efficacy assessment

Efficacy analyses were based on the subgroup population as the main analysis set. Some of the efficacy endpoints for the subgroup analysis were the same as those used in the individual RCTs. The primary endpoint of the current study was defined as the time from the end of surgery, defined as tracheal tube extubation, to their first bowel movement (FBM). The same definition was used in the previous three studies. In addition, a sensitivity analysis was performed to evaluate the reliability of the main result. The approach for the sensitivity analysis was identical to that used in the whole subgroup population, which was performed in subpopulations according to several clinical features, such as age, BMI, dosage, history, present illness, and surgery-related complications (i.e., anastomotic leakage). The results were compared with those of the subgroup population and reported for both the primary outcome and the sensitivity analyses.

### Statistical analyses

The primary endpoint was analyzed using survival analysis methods, including the Kaplan–Meier estimation, the log-rank test, and a Cox regression analysis. The log-rank test and Cox regression analyses were performed with stratification according to the organs that underwent surgery. When the time from tracheal tube extubation to the FBM could not be obtained due to withdrawal of participation in the clinical trial (*n* = 11), we excluded these subjects from the log-rank test and Cox regression analyses. To evaluate imbalances in background factors, the Cochran–Mantel–Haenszel test was used, again with consideration of differences according to the organ undergoing surgery. A *p* value < 0.05 was considered statistically significant. All statistical analyses were performed using SAS version 9.3 (SAS Institute, Cary, NC, USA).

## Results

Data from the 740 eligible patients, including 45.4%, 28.2% and 26.4% with colon, liver and gastric cancer, respectively, were pooled for the efficacy analysis sets. The subgroup population included 410 patients (DKT group, *n* = 214; placebo group, *n* = 196) who did not have a BM before the first diet after surgery [colon cancer, *n* = 152 (37.1%); liver cancer, *n* = 176 (42.9%); gastric cancer, *n* = 82 (20.0%)]. The mean duration of surgery was 4.2 h (range 1.2–12.6 h). Patients received an average of 13.56 g/day (range 0–15.0 g/day) DKT until their FBM.

A comparison of the main analysis group (*n* = 410) with all study subjects (*n* = 740) indicated that the proportions of patients differed slightly concerning the organs targeted for surgery. In the main analysis group, no significant differences in background factors were found between the DKT and placebo groups, and the dosages were also similar between the two groups (Table 1a). The DKT treatment group and placebo group were comparable in the main analysis group. In the non-main analysis group (*n* = 330), while no significant differences in background factors were found between the DKT and placebo groups, the dosages did differ between the groups (Table 1b).

**Table 1:** Background factors of the main analysis (a) and non-main analysis (b)

Factor	Placebo group	DKT group	Total	CMH test
(a) Main analysis group (group 1) *n* = 410 (colon: 152/liver: 176/stomach: 82)				
Sex				
Male	128 (65.3%)	138 (64.5%)	266 (64.9%)	*p* = 0.9933
Female	68 (34.7%)	76 (35.5%)	144 (35.1%)	
Age				
Mean	68.0	67.8	67.9	*p* = 0.8733
Range	28–88	28–88	28–88	
BMI (kg/m^2^)				
Mean	22.667	22.844	22.759	*p* = 0.8646
Range	15.04–39.06	14.61–42.10	14.61–42.10	
Coexisting diseases				
No	100 (51.0%)	115 (53.7%)	215 (52.4%)	*p* = 0.5060
Yes	96 (49.0%)	99 (46.3%)	195 (47.6%)	
Medical history				
No	99 (50.5%)	118 (55.1%)	217 (52.9%)	*p* = 0.4336
Yes	97 (49.5%)	96 (44.9%)	193 (47.1%)	
Surgery-related complications				
No	169 (86.2%)	179 (83.6%)	348 (84.9%)	*p* = 0.1837
Yes	27 (13.8%)	35 (16.4%)	62 (15.1%)	
ECOG performance status				
0	184 (93.9%)	197 (92.1%)	381 (92.9%)	*p* = 0.5566
1	12 (6.1%)	17 (7.9%)	29 (7.1%)	
Duration of surgery(min)				
Mean	253.6	244.9	249.1	*p* = 0.7238
Range	72–589	79–753	72–753	
Loss of blood volume at surgery(mL)				
Mean	458.9	513.6	487.5	*p* = 0.3163
Range	0–6180	0–6800	0–6800	
Average daily dose from 2 to 8d				
Mean	13.95	13.56	13.75	*p* = 0.2714
Range	0.0–15.7	0.0–15.0	0.0–15.7	
Average daily dose until defecation				
Mean	13.48	13.62	13.56	*p* = 0.4992
Range	0.0–15.0	0.0–15.0	0.0–15.0	
(b) Non-main analysis group *n* = 330 (colon: 184/liver: 33/stomach: 113)				
Sex				
Male	121 (72.9%)	113 (68.9%)	234 (70.9%)	*p* = 0.3712
Female	45 (27.1%)	51 (31.1%)	96 (29.1%)	
Age				
Mean	68.0	65.9	66.9	*p* = 0.2276
Range	35–91	37–88	35–91	
BMI (kg/m^2^)				
Mean	22.587	23.004	22.794	*p* = 0.4759
Range	15.57–31.12	15.20–37.22	15.20–37.22	
Coexisting diseases				
No	89 (53.6%)	100 (61.0%)	189 (57.3%)	*p* = 0.1479
Yes	77 (46.4%)	64 (39.0%)	141 (42.7%)	
Medical history				
No	100 (60.2%)	91 (55.5%)	191 (57.9%)	*p* = 0.4330
Yes	66 (39.8%)	73 (44.5%)	139 (42.1%)	
Surgery-related complications				
No	117 (70.5%)	128 (78.0%)	245 (74.2%)	*p* = 0.1004
Yes	49 (29.5%)	36 (22.0%)	85 (25.8%)	
ECOG performance status				
0	155 (93.4%)	154 (93.9%)	309 (93.6%)	*p* = 0.8470
1	11 (6.6%)	10 (6.1%)	21 (6.4%)	
Duration of surgery(min)				
Mean	223.0	227.0	225.0	*p* = 0.8393
Range	102–522	80–585	80–585	
Loss of blood volume at surgery(mL)				
Mean	308.8	330.3	319.5	*p* = 0.6205
Range	10–1400	10–3343	10–3343	
Average daily dose from 2 to 8d				
Mean	11.68	12.63	12.15	*p* = 0.0388
Range	0.0–15.0	0.0–15.0	0.0–15.0	
Average daily dose until defecation				
Mean	–	–	–	–
Range	–	–	–	

Values represent the number of patients (proportion in each group), mean, range, and *p* values of the CMH tests. The CMH tests were performed using table scores for discrete variables and rank scores for continuous variables. The presence of a surgery-related complication (“Yes”) was defined in cases of the development of at least one of following diseases: ileus, anastomotic leakage, intra-abdominal abscess, gastrointestinal motility disorder, abdominal distention, postoperative pancreatitis/pancreatic leak, or bacterial peritonitis. The common denominator in three different randomized controlled trials was the postoperative administration of DKT or placebo on days 2-8 (JFMC39-0902, 40-1001 and 42-1002)

*CMH* Cochran–Mantel–Haenszel test, *BMI* body mass index, *DKT* daikenchuto, *ECOG* Eastern Cooperative Oncology Group, *CI* confidence interval, *d* day, *JFMC* the Japanese Foundation for Multidisciplinary Treatment of Cancer

**Fig. 1:**
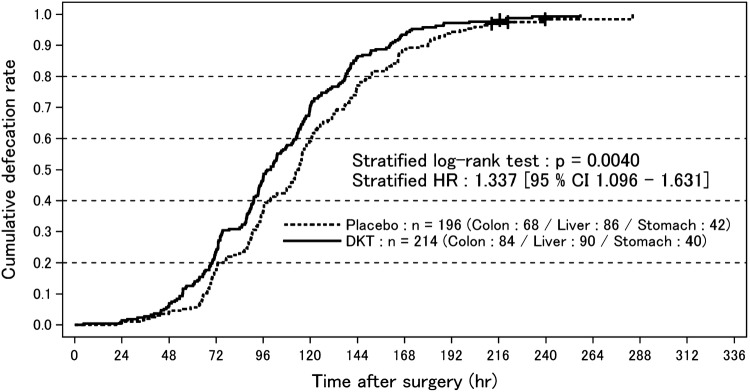
Cumulative defecation rate after surgery in the main analysis group. Curves represent the cumulative defecation rates of the Daikenchuto-treated and placebo-treated groups drawn using the Kaplan–Meier method. Censors are depicted using the “+” symbol on the curves. *DKT* daikenchuto

**Fig. 2:**
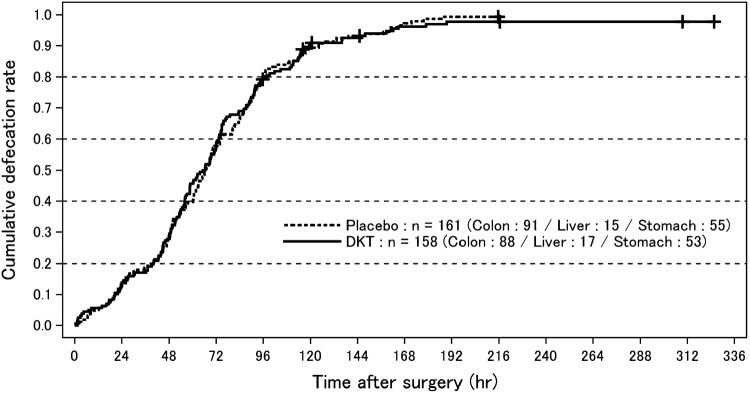
Cumulative defecation rate after surgery in the non-main analysis group. Curves represent the cumulative defecation rates of the Daikenchuto-treated and placebo-treated groups drawn using the Kaplan–Meier method. Censors are depicted using the “+” symbol on the curves. *DKT* daikenchuto

Our study revealed that there was a statistically significant difference in the primary endpoint between the DKT and placebo groups in the main analysis cohort [stratified log-rank test; *p* = 0.004; stratified hazard ratio (HR) 1.337; 95% confidence interval (CI) 1.096–1.631; Fig. 1]. In contrast, no significant difference in the primary outcome was noted between the 2 groups in the non-main analysis group (stratified log-rank test; *p* = 0.6322; stratified HR 0.947; 95% CI 0.756–1.185; Fig. 2). The analysis including all study subjects showed that there was also no significant difference in the primary outcome between the two groups (stratified log-rank test; *p* = 0.1016; stratified HR 1.131; 95% CI 0.976–1.312).

In the main analysis group, the median time from extubation to the FBM was 113.84 h (95% CI 107.0–117.03) in the placebo group (*n* = 194) and 99.09 h (95% CI 93.75–106.17) in the DKT treatment group (*n* = 214), which was 14.75 h shorter than that in the placebo group. The median time from extubation to the first meal was 66.5 h (interquartile range 43.0–90.9) in the placebo group and 63.5 h (interquartile range 40.2–89.8) in the DKT treatment group. The analysis including the non-main analysis subjects found that the median time from extubation to the FBM was 66.40 h (95% CI 60.67–71.15) in the placebo group (*n* = 158) and 65.81 h (95% CI 57.15–72.00) in the DKT treatment group (*n* = 161), both of which were significantly shorter than the respective values in the main analysis group.

Sensitivity analyses were performed using the following six subpopulations: age < 75 years old (*n* = 295), BMI < 30 kg/m^2^ (*n* = 393), no medical history (*n* = 217), no complications at the time of enrollment (*n* = 215), no surgery-related complications (*n* = 348), and an average daily DKT dose of ≥ 10 g until the FBM (*n* = 369) (Table 2). Statistically significant differences were observed in all subpopulations. In addition, there were considerable increases in the HRs in all subpopulations, when the absence of surgery-related complications (*n* = 62; 16 ileus, 29 gastrointestinal motility disorders, 40 abdominal distension, 4 anastomotic leakage, 5 intra-abdominal abscess, 3 pancreatic fistula, and 3 pancreatitis) was excluded as a variable.

**Table 2:** The results of the sensitivity analysis in the main analysis

Factor	Number of patients	Stratified log-rank test	Stratified hazard ratio (95% CI)
Whole main analysis population	*n* = 410	*p* = 0.0040	1.337 (1.096–1.631)
Subset			
Age < 75 years	*n* = 295	*p* = 0.0044	1.406 (1.111–1.779)
BMI < 30 kg/m^2^	*n* = 393	*p* = 0.0029	1.359 (1.110–1.665)
No medical history	*n* = 217	*p* = 0.0025	1.569 (1.169–2.104)
No coexisting diseases	*n* = 215	*p* = 0.0229	1.376 (1.044–1.814)
No surgery-related complications	*n* = 348	*p* = 0.0101	1.327 (1.069–1.647)
Average daily dose until defecation ≥ 10 g	*n* = 369	*p* = 0.0019	1.395 (1.130–1.723)

Values represent the number of patients, *p* values of stratified log-rank tests, and stratified hazard ratios in the Daikenchuto-treated versus placebo-treated groups, along with 95% confidence intervals for the whole subgroup population and all subpopulations used in the sensitivity analysis

*BMI* body mass index, *CI* confidence interval

## Discussion

In the present study, DKT significantly improved the prolonged POI after major OAS in the main analysis group. The number of patients in the subgroup group (*n* = 410) included 152, 176, and 82 patients with colon, liver, and gastric cancer, respectively. Sufficient statistical power of the pooled analysis including 410 patients was not achieved in the individual RCTs due to the relatively small number of patients.

The common primary endpoint of the RCTs pooled in this study was the time to the FBM after the completion of surgery [10–12]. However, RCT results did not show an appreciable reduction in time to initial defecation provided by DKT, as anticipated. The authors speculate that a potential confounder masked the effect of DKT and hence its effect on the POI. DKT components are known to stimulate intrinsic intestinal motility factors (e.g., neurotransmitters, among others), but they do not act like neurotransmitters by themselves [7, 8]. It is possible that, in the group that recovered earlier from the physiological ileus state, endogenous intestinal motility factors were already at work, precluding the effect of DKT. Therefore, excluding the patients who recovered from physiological POI and those who did not require medical treatment resulted in a group of patients with a high possibility of morbid ileus expression; these patients were used as the main analysis group in this study. The purpose of this study was to verify the true pharmacological action of DKT.

Although the end point of POI is controversial, the occurrence of a BM appears to be the most reliable endpoint [1, 3]. Nearly half of the patients in the present study experienced a BM before the first diet after surgery. These patients were therefore deemed to have recovered from physiologic POI (low incidence of pathologic POI) provided recurrent POI did not occur. The median time from surgery to the FBM for these patients was 66.4 h (95% CI 60.7–71.2) in the placebo group and 65.8 h (95% CI 57.2–72) in the DKT group. These median times with 95% CIs are defined as the category of physiologic POI. Therefore, to evaluate the effect of DKT on pathologic POI more precisely, we excluded such low-risk patients from the main analysis population.

The main analysis population did not experience a BM before the first diet after surgery. A diet is given to patients after surgery according to the comprehensive clinical judgment of doctors based on abdomenal X-ray findings, flatus, and bowel sounds, even if there is no BM. Early feeding of patients who had undergone abdominal surgery led to a reduction in the length of the hospital stay, improvement of the metabolic status, a reduced rate of septic complications, and a reduced morbidity rate [15]. However, the incidence rate of prolonged POI following major abdominal surgery was 10–25% [16]. Furthermore, prolonged POI increases the length of the hospital stay [16].

We hypothesized that patients who do not have a BM before their first diet after surgery would be a suitable group for evaluating the effect of DKT on pathologic POI after OAS in the present subgroup analysis. Our study of the main analysis group showed that the median time from surgery to the FBM was 114 h (95% CI 107.0–117.03) in the placebo group and 99 h (95% CI 93.75–106.17) in the DKT treatment group. Both the median time and 95% CI fall outside the physiologic POI category and are likely to fall into the category of prolonged POI. These results support our hypothesis that the main analysis population is at risk of prolonged POI and requires medical treatment to recover.

The three RCTs included in the present study enrolled patients with three different cancers who underwent OAS that targeted different organs. These three different types of OAS are typical stressors and causes of pathological POI, in general. Therefore, the results from one RCT cannot be generalized to patients undergoing OAS for other organs. However, the data in the current study included patients with cancers of three type of OAS; therefore, our findings are more generalizable than those from a study on a single type of OAS. Although the differences in the organs were statistically weighted in the present study, a larger, high-quality RCT evaluating the efficacy of DKT should be conducted in the same organ, and the surgical procedures should also be improved.

Several important neurally mediated mechanisms have been suggested as mediating the increased effective intestinal motility of DKT. One proposed mechanism posits that DKT accelerates the acetylcholine release from cholinergic myenteric neurons mediated by the activation of 5-HT receptors (5-HT3 and 5-HT4) [17, 18], and smooth muscles then contract due to the released acetylcholine through the stimulation of muscarinic receptors (M2R and M3R). Alternatively, it has been reported that DKT increases the plasma levels of motilin, a gastrointestinal polypeptide hormone, which improves morphine-induced constipation in humans and canines [19, 20]. As another potential mechanism, DKT may induce the release of substance P from primary sensory nerves through the transient receptor potential vanilloid 1 on intramucosal terminal sensory nerves, which contracts smooth muscle [21, 22]. DKT may also induce the release of serotonin through the transient receptor potential ankyrin 1 on enterochromaffin cells, resulting in coordinated peristaltic motility in the small intestine [23]. As a final potential mechanism, DKT ingredients may accelerate colonic motility by inhibiting the two-pore-domain potassium channel subfamily K (KCNK3 and KCNK9) in intestinal smooth muscle and neuronal cells [24]. These lines of evidence indicate that while DKT ingredients themselves are not neuronal transmitters or neuropeptides, DKT can stimulate the release of neuronal transmitters and gastrointestinal hormones for BMs [7], mainly via the modulation of the peripheral neural machinery in the intestinal wall.

We selected subpopulations for the sensitivity analysis based on the absence of postoperative complications, medical history, and coexisting disease, all of which are considered to be associated with recovery from POI. In addition, we used the age and BMI as additional variables for the subpopulations. Previous pharmacokinetic studies on DKT showed that the age and BMI were significantly and inversely correlated with the absorption of active DKT ingredients [14]. We also used the dosage as a variable for the sensitivity analysis, which showed that the absorption rate of active DKT ingredients was dose-dependent. The HRs tended to rise in subpopulations of patients who were < 75 years of age, who had a BMI < 30 kg/m^2^, and who were receiving more than two-thirds of the expected DKT dose.

Population pharmacokinetic models for DKT using data from two RCTs on DKT pharmacokinetics were developed in Japan and the United States [14]. Participants received single oral doses of 2.5, 5, or 10 g DKT. Active DKT ingredients were effective for BMs in a dose-dependent manner and inversely correlated with the BMI and age, and ethnic differences between the Japanese and US participants were minimal. An analysis of the BMI revealed that a nearly twofold increase in the BMI from 18 to 30 kg/m^2^ was associated with a twofold decrease in the plasma concentrations of active DKT ingredients. We also found that the age was a covariate, with a threefold increase in age from 20 to 60 years being associated with a 1.5-fold decrease in plasma concentrations of active DKT ingredients.

In the current study, the ranges of the BMI and age in the main analysis subgroup were 14.6–42.1 kg/m^2^ and 28–88 years, respectively. Therefore, an evaluation of the outcomes adjusted by the concentrations of active ingredients of DKT should be conducted with consideration of the patient BMI and age in future RCTs.

Traditional Japanese medicines, such as DKT, were developed from 500 to over 1500 years ago [8, 25]. Given that the average life expectancy was shorter and the average weight lower at that time than at present, the differences in the blood concentrations of active DKT ingredients with different doses were not likely to be significant. The use of DKT is mentioned in a famous classical book of traditional Japanese medicine (Jin Gui Yao Lue) [26]. In that book, DKT is said to be more effective in slim people than in heavier-set people. This statement matches well with the results of the present study concerning patients with a BMI < 30 kg/m^2^.

### Limitations

Several limitations associated with the present study warrant mention. First, the subgroup analysis might be confounded by differences in surgical procedures owing to differences in the cancer types and target organs among the three RCTs. Second, the occurrence of pathologic POI was related to multiple factors, including the surgical procedure, experience of the surgeon, tissue quality, hospital environment, total parenteral nutrition, and additional medical therapies. As a result, the database was somewhat imprecise. To address this type of heterogeneity, larger, high-quality RCTs that evaluate the efficacy of DKT should be conducted, and more detailed analyses, such as analyses of financial constraints of DKT, must be performed to strengthen the reliability of any conclusions.

## Conclusion

This subgroup analysis of three RCTs revealed that DKT significantly accelerated the POI recovery in patients at risk of pathologic POI occurrence after major OAS compared with the placebo group. Our findings suggest that the benefits of DKT might be enhanced in patients who are younger than 75 years of age, those with a BMI < 30 kg/m^2^, and those receiving a daily DKT dosage of ≥ 10 g.

## Abbreviations

POI: Postoperative ileus
OAS: Open abdominal surgery
DKT: Daikenchuto
RCT: Randomized controlled trial
JFMC: The Japanese Foundation for Multidisciplinary Treatment of Cancer
BMI: Body mass index
HR: Hazard ratio
CI: Confidence interval
BM: Bowel movement
FBM: First bowel movement
